# The role of generalized trust in COVID-19 vaccine acceptance

**DOI:** 10.1371/journal.pone.0278854

**Published:** 2022-12-22

**Authors:** Philipp Simon Eisnecker, Martin Kroh, Simon Kühne

**Affiliations:** Faculty of Sociology, Bielefeld University, Bielefeld, North Rhine-Westphalia, Germany; University of Belgrade Faculty of Organisational Sciences: Univerzitet u Beogradu Fakultet organizacionih nauka, SERBIA

## Abstract

Immunization by vaccination is one of the most important tools for fighting the COVID-19 pandemic. Yet in many countries, immunization campaigns have been hampered by vaccine hesitancy within the population. Building on the idea that vaccination decisions are embedded in the broader societal context, we study the role of generalized trust—the belief that most people can generally be trusted—in vaccine acceptance. Immunization campaigns face an inherent collective action problem: As all individuals benefit collectively from high immunization rates regardless of individual contribution, especially those with a low risk of severe COVID infection have an incentive to decide against the (perceived) costs and risks of vaccination. We argue that generalized trust may help to overcome this problem by encouraging the belief that cooperation for the common good is achievable and that those who cooperate are unlikely to be exploited by others. We further argue that the positive effect of generalized trust on vaccination decisions is weaker among individuals who are at higher risk of severe outcomes from the disease, as the collective action problem is less pronounced in this group. To test our predictions, we used data from the SOEP-CoV survey, which queried a representative probability sample of Germany’s population between January and February 2021 on topics connected to the pandemic. Using multiple logistic regression models, and in line with expectations, we found a positive and robust link between generalized trust and the willingness to accept vaccination as soon as offered. However, overall, our examination of heterogeneous effects does not unequivocally support the idea that the role of generalized trust varies according to individual COVID risk.

## Introduction

As of January 2022, there were 319 million confirmed cases of coronavirus disease (COVID-19) caused by the SARS-CoV-2 virus worldwide, including 5.5 million deaths [1]. The World Health Organization declared the COVID-19 outbreak to be a global pandemic in March 2020 [2].

Safe and effective vaccines are seen by many experts as a way out of the pandemic [3]. Beginning in December 2020, the EU Commission granted several vaccines conditional marketing authorization [4], and the US Food and Drug Administration authorized a number of vaccines for emergency use [5]. At a time when many of the poorest countries still had virtually no access to vaccines [6], many wealthy countries launched mass vaccination campaigns but often encountered decreasing vaccination rates and high vaccine hesitancy in parts of their populations. As of January 2022, so roughly one year after vaccines became available at a large scale, only 72 percent of the population in Germany [7], 71 percent in Austria [8], 70 percent in the United Kingdom [9], and 63 percent in the United States [10] were fully vaccinated. The WHO highlighted vaccine hesitancy—the reluctance or refusal to vaccinate despite the availability of vaccines—as one of ten threats to global health [11] even before the current pandemic.

Vaccine hesitancy is understood as existing along a spectrum between actively seeking and completely refusing vaccination [12]. The spectrum includes different groups. For example, some are willing to accept only certain vaccines, some choose to delay vaccination, and some get vaccinated but have second thoughts about it. Scholars from various disciplines including medicine (e.g., [13]), public health (e.g., [14]), psychology (e.g., [15]), and sociology (e.g., [16]) have provided valuable insights into a comprehensive understanding of the causes, dynamics, and consequences of vaccine hesitancy.

Trust has been pointed out as central factor in vaccine decision-making [17]. The role of trust has been researched in relation to vaccines themselves, to the providers of vaccines, the policy makers involved in approving and recommending vaccines, and the media distributing information about vaccines [17, p. 1600]. However, social scientists have pointed out that vaccine hesitancy is embedded in specific historical, political, and socio-cultural contexts and should be studied as such [12, p. 1765].

In this paper, we therefore move away from the topic of trust in the elites, experts, and institutions that are directly linked to vaccines to look at trust in the wider societal context and people within it. Specifically, we are interested in the role of generalized trust, which extends beyond people known from face-to-face interactions and includes others about whom the individual has no direct information. Generalized trust is the idea that, in general, most people can be trusted [18, p. 2].

Based on the theoretical underpinning that reaching high immunization rates within a community constitutes a collective action problem [19], we put forward two expectations: First, as generalized trust may be one way to overcome the collective action problem on the individual level, we expect that individuals’ vaccine acceptance and generalized trust are linked positively. Existing empirical evidence on this link is contradictory [19–27]. Second, we expect that this link is weaker or stronger depending on the individual’s COVID risk, that is, the individual’s benefit from vaccination. We are not aware of studies that have tested this heterogeneous effect so far. By testing these two expectations on a solid theoretical foundation of research on the collective action problem, we aim to contribute to the literature on the predictors of vaccine hesitancy.

To examine our two expectations, we employ data of the second wave of the SOEP-CoV survey [28]. The survey was administered to a representative sample of the German population between January and February 2021 on topics related to the pandemic, including COVID infections and testing, vaccine hesitancy, general health status, and generalized trust.

In the following, we first discuss the role of trust in the collective action problem of vaccination. Then, we introduce the SOEP-CoV survey, describe how we operationalized our concepts, and lay out our analytical methods. After presenting our results, we provide a summary and discussion.

## Trust and the collective action problem of vaccination

The decision to receive or refuse a particular vaccine is made in relation to the subjective benefits and costs of vaccination. Subjective benefits are large if the likelihood of infection is estimated as high and the disease is seen as severe, while the vaccine is deemed effective in preventing infection and severe illness. Subjective costs encompass monetary costs, as well as the time and effort to get vaccinated [29]. Additionally, subjective costs include the expectation of frequent and serious side effects of vaccination. Indeed [14], reported that the most important reason given for vaccine refusal were safety concerns. Both benefits and costs are often connected to different aspects of trust, or the lack thereof.

Although the term “trust” is frequently invoked in the vaccination literature, it often remains undertheorized or undefined [17, p. 1602]. Unfortunately, there is no agreement on the definition of trust in the social sciences [30, p. 414]. Building on ideas of Georg Simmel [31], (see also [32]) defines trust as a mental process that begins with an interpretation of reality. This interpretation then gives “good reasons” that lead to an expectation of favorable (in the case of trust) or unfavorable (in the case of distrust) outcomes—the final stage of the trust process. However, as knowledge is always incomplete, the gap between interpretation and expectation must be overcome by a “leap of trust.” As the process of modern vaccine development is obscure for non-experts, this leap of trust in a vaccine must be made based on incomplete knowledge about its side-effects and effectiveness. This leap is likely even larger for the COVID-19 vaccine, with which patients have no prior experience and which is based on novel scientific principles.

Vaccine acceptance is related to different aspects of trust [17, p. 1600]. Research shows that trust directly related to vaccines and the systems involved in their development and distribution heightens vaccine acceptance: Beliefs that vaccines are safe [13, 14, 22, 33, 34] and effective [13, 14, 29, 34] are positively connected to vaccine acceptance, as is trust in the health care system [14, 15, 19] and in science and research [35–37]. Trust in the media is an important transmitter for all kinds of trust relationships and as such is also positively linked to vaccine acceptance [33, 38, 39].

However, vaccine hesitancy is always embedded in a broader societal context [12, p. 1763], for which far less research is available. Trust is an important element of this broader context, and one of these “external levers of trust” is generalized trust [17, p. 1600]. In contrast to particularized trust, which relates to a particular person or system (e.g., a doctor or the health care system) and a particular domain (e.g., vaccine distribution), generalized trust encompasses all people (including strangers) and is not restricted to a specific domain [30, p. 414]. Generalized trust is an optimistic and relatively stable worldview according to which most people can be trusted, based on a foundation of common values [40]. A typical survey question measuring generalized trust is “Generally speaking, would you say that most people can be trusted or that you can’t be too careful in dealing with people?”. This item has been included in major national and international surveys including the American General Social Survey [41], the European Value Study [42], and the European Social Survey [43].

Benefits from vaccination lie both on the individual (e.g., reduced risk of own infection and severe illness) and the societal (e.g., reduced strain on health services) level. While everyone can enjoy the societal benefits relatively equally, the strength of the individual benefits varies depending on how threatening the disease appears to the individual. Public health messaging during the pandemic has emphasized that certain factors such as high age and preexisting medical conditions strongly increase the risk of severe COVID disease and mortality [44]. By contrast, for individuals with a low personal COVID risk, the benefits of vaccination lie mainly on the societal level.

Achieving high immunization rates among such individuals can be described as a collective action problem ([19, p. 854]; see [45], for the original formulation of the problem): Individuals are incentivized to “free-ride” on the benefits connected to an immunized population rather than shouldering the (perceived) risks and costs of vaccination themselves. This may lead to “tragedy of the commons” situations, in which individuals pursue their own self-interest by refusing vaccination and create outcomes that are detrimental to the common good of disease control in an immunized population [46, p. 8]. Reaching high vaccination rates on a national or even global level can further be characterized as a large-scale collective action problem [47] involving a large number of individuals distributed over vast areas who are typically anonymous to each other and thus lack accountability. Therefore, classic solutions to the collective action problem that rely, for instance, on communication between individuals [48], reciprocity [49], or punishment of free-riders by other group members [50] are less feasible [47]. Considering this, it not surprising that some [51] argue that the collective action problem of mass vaccination should be resolved by an external authority such as the state imposing vaccine mandates. However, such mandates may be politically or legally infeasible in many countries.

In the absence of an external authority, trust is frequently discussed as a solution to collective action problems [52]. In experimental research settings, trust has been found to heighten cooperation between two or a small number of test subjects, often within the context of repeated games that provide the possibility for reciprocity [53]. The trust investigated in these kinds of experimental (or similar real-world) settings can be described as particularized trust in specific persons within a specific environment. As mass vaccination is a large-scale collective action problem involving numerous individuals and anonymity, we argue that instead of particularized trust, individuals’ generalized trust may offer a way to address this collective action problem. We expect generalized trust to play an especially pronounced positive role in vaccine acceptance among low-risk groups, as their individual benefits from vaccination are relatively low.

Generalized trust has been described as a “potential readiness of citizens to cooperate with each other and the abstract preparedness to engage in civic endeavors with each other” [54, p. 397]. People who are trusting believe that others—including strangers—are generally well-motivated [40, p. 572] and will likely not take advantage of them. To trusting people, cooperation makes sense from a strategic point of view: Since others can be expected to participate with them in vaccination efforts, the common good of high immunization rates is achievable. People who are less trusting, in contrast, have more cynical views of others and may expect them to free-ride. Consequently, to avoid being taken advantage of, they do not participate in collective efforts either.

Empirical evidence of the expected positive link between generalized trust and vaccine acceptance is mixed: [19] found a positive relationship between generalized trust and acceptance of vaccination against the 2009 A(H1N1) pandemic in a Swedish sample. Likewise, [21] report a positive link between generalized trust and trust in the influenza vaccine in their US sample. Among the studies that have investigated the role of generalized trust in the COVID pandemic, [20, 22, 23] found no relationship between trust and COVID vaccine acceptance [24–27], found a positive one.

Free-rider problems exist if individuals can benefit from a good without having to contribute to it themselves. This problem can be alleviated if a separate incentive, in addition to the benefit from the public good, is offered on the condition that the individual contributes to the public good [45, p. 2]. For individuals at risk from severe COVID infection, the individual benefits from vaccination are such a separate incentive. Contrary to the public good of high societal immunization rates, the individual benefit of vaccination is not subject to the free rider problem. Therefore, generalized trust can be expected to play a comparatively smaller role in vaccine acceptance for high-risk groups. To our knowledge, research on these potential heterogeneous effects of generalized trust on vaccine acceptance is lacking.

## Data and methods

### The SOEP-CoV survey

To test our predictions, we used data from the SOEP-CoV survey [28] conducted as part of the German Socio-Economic Panel (SOEP; see [55]) study. The SOEP is a longitudinal annual standardized survey of representatively selected German households. Running since 1984 (since 1990 in Eastern Germany), approximately 30,000 adult respondents in 15,000 households are currently surveyed by the SOEP each year. The interviews with participants of the SOEP study and the further processing of the data are consent-based. Before participating, respondents receive a data protection sheet which informs them about the purposes and scope of data collection, about the group of people authorized to access the data, and about their data privacy rights. The interviewer then asks them whether they have read the contents and whether they are willing to take part in the survey in the knowledge of the contents. The interview only commences after respondents have verbally answered “yes” to both questions. This is technically ensured by making the interviewers check one box for each question in the computer software. The survey institute ensures that the interviewers’ declarations are personalized and stored separately from the survey data. The manner of storage ensures that the granting of informed verbal consent by the respondents can be permanently proven. The SOEP employs a wide catalogue of repeated question modules with a focus on socio-economic topics, although modules targeting current developments are frequently added. The SOEP-CoV project is an add-on to the regular SOEP. The Ethics Committee of Bielefeld University has reviewed the study under the application number 2021–293 of 2021/12/10 according to the ethical guidelines of the German Association of Psychology (Deutsche Gesellschaft für Psychologie; DGPs), which correspond to the guidelines of the American Psychological Association (APA). Based on the submitted materials, the Ethics Committee of Bielefeld University approved of the study, as described, as ethically appropriate. A subsample of adult 6,694 SOEP respondents were recruited for a first telephone interview between March 31 and July 4, 2020, during the first wave of the pandemic in Germany. Questions targeted attitudes and behaviors relating to the COVID-19 pandemic, as well as possible socio-economic predictors and consequences of these attitudes and behaviors. A second telephone interview with participating households from the first wave was conducted between January 18 and February 15, 2021, and 6,038 of the first-wave respondents took part. SOEP and SOEP-CoV represent a unique database to study the factors that influence the short-, medium-, and long-term socio-economic consequences of the coronavirus in Germany. In contrast to other (nonprobability) ad-hoc surveys on the topic, the SOEP data provide detailed information about individuals and households prior to the pandemic, thus increasing the potential to study causal pandemic effects.

The second-wave interviews employed in our analyses were conducted when Germany had just gone through its second and most devastating COVID wave up to that point in terms of both case numbers [56] and deaths [57]. Vaccination in Germany had started on December 31, 2020, although vaccine supplies were limited and only available to individuals in the highest priority group (those older than 80, residents of nursing homes, and frontline healthcare workers). By the end of February 2021, around 4 million of Germany’s 83 million residents had received their first shot [7]. Survey respondents were therefore asked about their vaccine acceptance during a period in which several vaccines had already been developed and approved, although it remained unclear to the vast majority of the population when they would actually receive them. Additionally, it should be noted that recommended vaccines are free of charge in Germany and that expected direct monetary costs are therefore highly unlikely to influence vaccine acceptance.

### Variable operationalization and modeling approach

#### Dependent variable

During the second SOEP-CoV telephone interview, respondents were asked if they had been vaccinated yet against COVID. Those answering “no” were asked if they would get vaccinated if they were offered vaccination, to which they could answer “yes, as soon as I am offered vaccination,” “yes, but I will wait a little longer,” “I am undecided if I want to get vaccinated,” and “no.” The binary dependent variable for the following analyses distinguishes between 1 (accepting) and 0 (hesitant). Those who were already vaccinated or who chose “as soon as offered” were defined as “accepting.” Following [11], according to which vaccine hesitancy is defined as “the reluctance or refusal to vaccinate despite the availability of vaccines,” respondents who were waiting, undecided, or unwilling to get vaccinated were defined as “hesitant.”

#### Independent variables

Generalized trust was operationalized using two survey questions that the SOEP group developed based on a survey item employed in the General Social Survey and the World Values Survey and that has been shown to be reliable and valid [58]. Respondents were asked to rate their agreement with the statements, “People can generally be trusted,” and “You can’t rely on anyone nowadays,” on a scale from 1 (“Totally agree”) to 4 (“Totally disagree”). From these two survey items, a single index was constructed in two steps: first, the first survey item was recoded to be oriented toward higher trust, now ranging from 1 (“Totally disagree”) to 4 (“Totally agree”). A test of the internal consistency of the items suggests a Cronbach’s alpha value of 0.62 for the sample studied in the following sections. Second, the arithmetic mean of both items was calculated for each respondent. Subsequently, the single trust index ranges from 1 (lowest trust) to 4 (highest trust).

In our analysis, we understand the risk of a severe COVID infection as the individual benefit that comes with being vaccinated. We employed two measures of this risk: subjectively perceived and officially defined COVID risk. For subjective COVID risk, respondents were asked to rate their risk of a life-threatening COVID infection within the next 12 months on a scale of 0 to 100 percent.

We defined official COVID risk in line with estimates by the national health authorities in Germany. These estimates were based on several individual characteristics such as age and health conditions and were used, for instance, to define the priority groups for individual vaccination [59]. Individuals were either placed into one of five priority groups with earlier access to vaccination or into a sixth group with no priority access. Based on available survey information, we replicated this risk prioritization: Respondents are categorized as at risk if they are 60 and older or if they are younger and suffer from dementia, obesity (BMI > = 30), heart disease, diabetes, cancer, hypertension, or asthma either in the present or at some time in the past. Respondents are categorized as not at risk if they are younger than 60 and suffer or suffered from none of these health conditions.

We chose to model official COVID risk based on the governmental priority list for two reasons: First, the plan largely reflects research on COVID risk factors known at that time (for a meta-analysis, see [44]). Second, the priority list was widely known and was the subject of considerable public debate in Germany. As a result, many people gained a broad understanding of their risk of severe COVID infection through the characteristics used in this list.

#### Control variables

Controls are based on previous studies on vaccine acceptance: we include gender and having a migration history (not born in Germany or parents not born in Germany), as being female [13, 14, 16, 20, 29, 33, 34, 38] and having a non-majority background [13, 14, 34, 36, 38] are often negatively connected to acceptance. We also control for marital status, education, household net equivalent income, and occupational status, because being married [60], having a higher education [15, 19, 20, 29, 34, 36], a higher socioeconomic status [13, 14, 20], and not being employed or self-employed [33, 36] were found to be positively connected to vaccine acceptance. With political attitudes often being associated with vaccine acceptance [14, 16, 20, 36, 38, 61], we also control for political left-right self-placement. Furthermore, we control for satisfaction with the federal government’s crisis management (on a scale from 0–10) as stand-in for governmental trust, which is connected positively to acceptance [62]. As media consumption is connected to vaccine acceptance [36, 39], a corresponding control variable is used. Respondents were asked where they obtained their information on COVID and, based on their answers, were categorized as using traditional media sources only (TV, radio, and newspapers), Internet only (social media and Internet searches), or mixed sources. Older persons are often more vaccine accepting [15, 16, 19, 20, 29, 34]. We therefore control for age in years in most models. Age was not controlled for in models including the indicator for official COVID risk, as the latter is partly based on age and the inclusion of an additional age variable would lead to multicollinearity issues.

#### Analytic strategy

As the dependent variable “vaccine acceptance” is binary, we employed multiple logistic regression analysis. To make the results more comparable between models, we present them as marginal effects. All descriptive illustrations and analyses are weighted using cross-sectional weights provided by the SOEP group [63].

## Results

### Descriptive

After deletion of cases with missing values for one or more variables (12 percent of all 2021 respondents), the sample consisted of 5,341 respondents. On the one hand, a clear majority of respondents (65 percent, see Table 1) were defined as “vaccine-accepting.” The group contains a very small subset of 4 percent who were already vaccinated at the time of the survey and 96 percent who were not yet vaccinated but who wanted to be vaccinated “as soon as offered.” On the other hand, this means that around 35 percent of respondents were vaccine-hesitant. This group consists of three subgroups that are roughly equal in size: those who wanted to get vaccinated but wanted to wait a little longer (12 percent), those who were unsure (12 percent), and those who did not want to get vaccinated (11 percent). The index of generalized trust takes a mean of 2.9, which falls slightly above the middle of the possible index range. With an average of 23 percent, respondents rated their risk of a life-threatening COVID infection within the next year as extremely high. This high value for subjective COVID risk may partly be explainable by the timeframe of the survey, which took place in the aftermath of Germany’s most devastating COVID wave so far. Around 64 percent of respondents are at heightened risk from COVID according to their vaccine prioritization, leaving 36 percent not at heightened risk.

**Table 1:** Variable distribution.

	Mean	Standard deviation
**Vaccine acceptance**	0.65	
**Generalized trust**	2.93	0.60
**Subjective COVID risk:** life-threatening infection (percent)	22.84	20.34
Official COVID risk*: prioritization for vaccination	0.64	
**Female**	0.50	
**Age**	56	18
**Migration history**	0.21	
**Marital status**		
Never Married	0.26	
Married/Civil union	0.54	
Divorced/Widowed	0.20	
**Education**		
Max. lower secondary edu.	0.31	
Intermediate secondary edu.	0.29	
Upper secondary edu.	0.13	
Tertiary edu.	0.27	
**Household net equivalent income**	2107	2076
**Occupational status**		
Working, full-time	0.38	
Working, not full-time	0.20	
Not working	0.42	
**Political leaning**		
Left	0.35	
Center	0.44	
Right	0.21	
**Satisfaction fed. government**	5.70	2.36
**Information source COVID**		
Only traditional media	0.38	
Mixed	0.52	
Only Internet	0.10	
N	5341	

Data: SOEP-CoV survey. Own calculations, weighted.

*See S1 Table for details.

### Bivariate analyses

In a first step to assess the relevance of generalized trust and its potential interaction terms for vaccine acceptance, we employed bivariate analyses.

**Fig 1:**
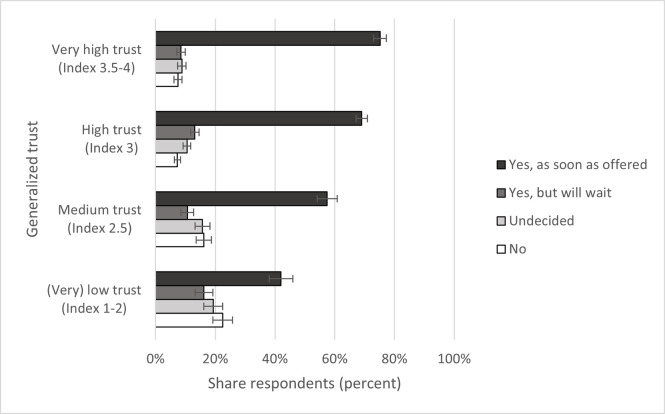
Generalized trust and vaccine acceptance. Data: SOEP-CoV survey. Own calculations, weighted. 95% confidence intervals.

Vaccine acceptance is strongly and positively connected to generalized trust (Fig 1): For example, 75 percent of those expressing very high trust (3.5–4 on trust index) claimed that they wanted the vaccine “as soon as offered,” which was the case for only 42 percent of those expressing (very) low trust (1–2 on trust index). At the other end of the spectrum, 22 percent of respondents indicating (very) low trust answered “no” to the question of vaccine acceptance compared to 16 percent indicating medium trust (2.5 on trust index) and around 7 percent indicating (very) high trust (3–4 on trust index).

**Fig 2:**
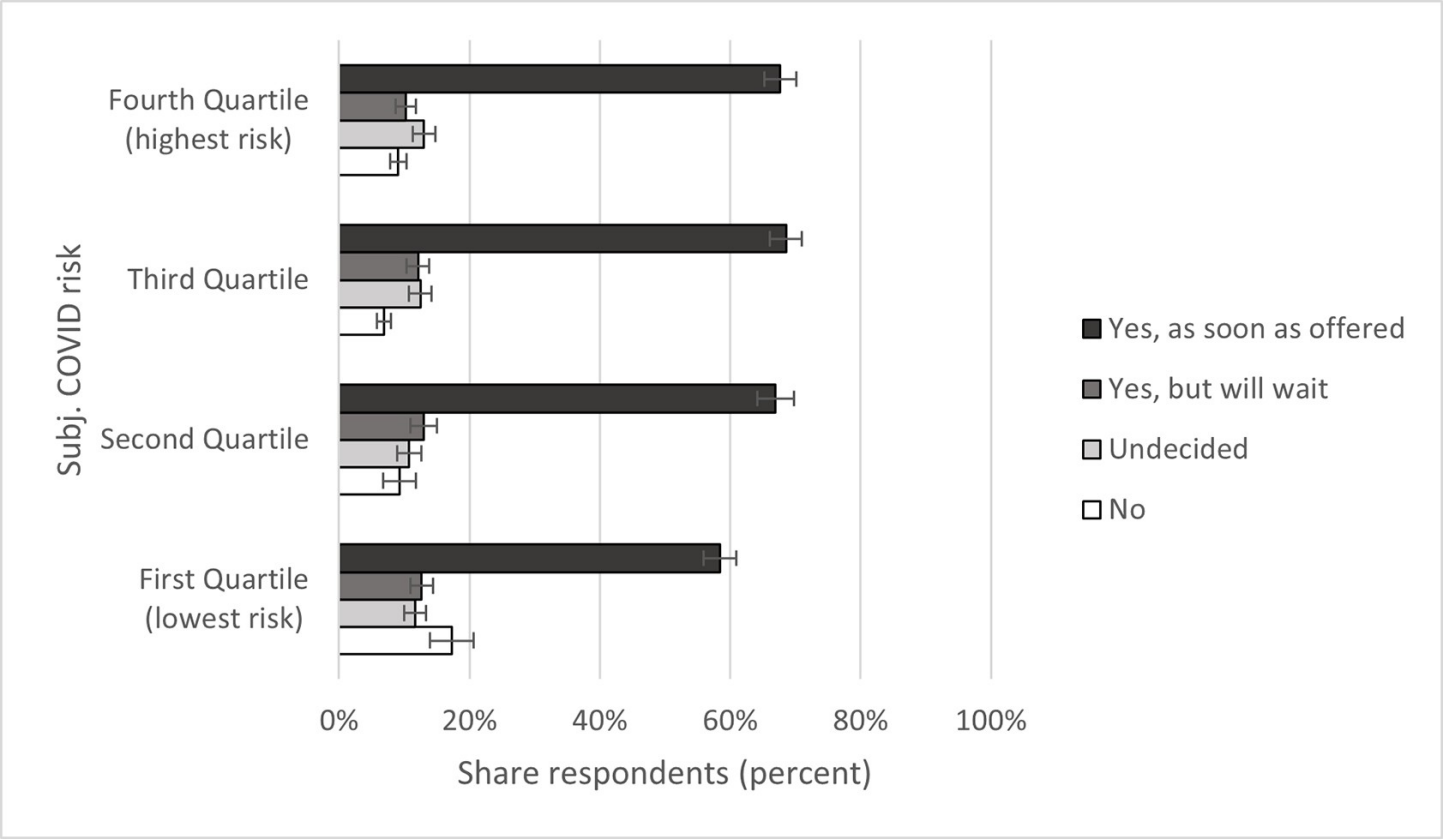
Subjective COVID risk and vaccine acceptance. Data: SOEP-CoV survey. Own calculations, weighted. 95% confidence intervals.

**Fig 3:**
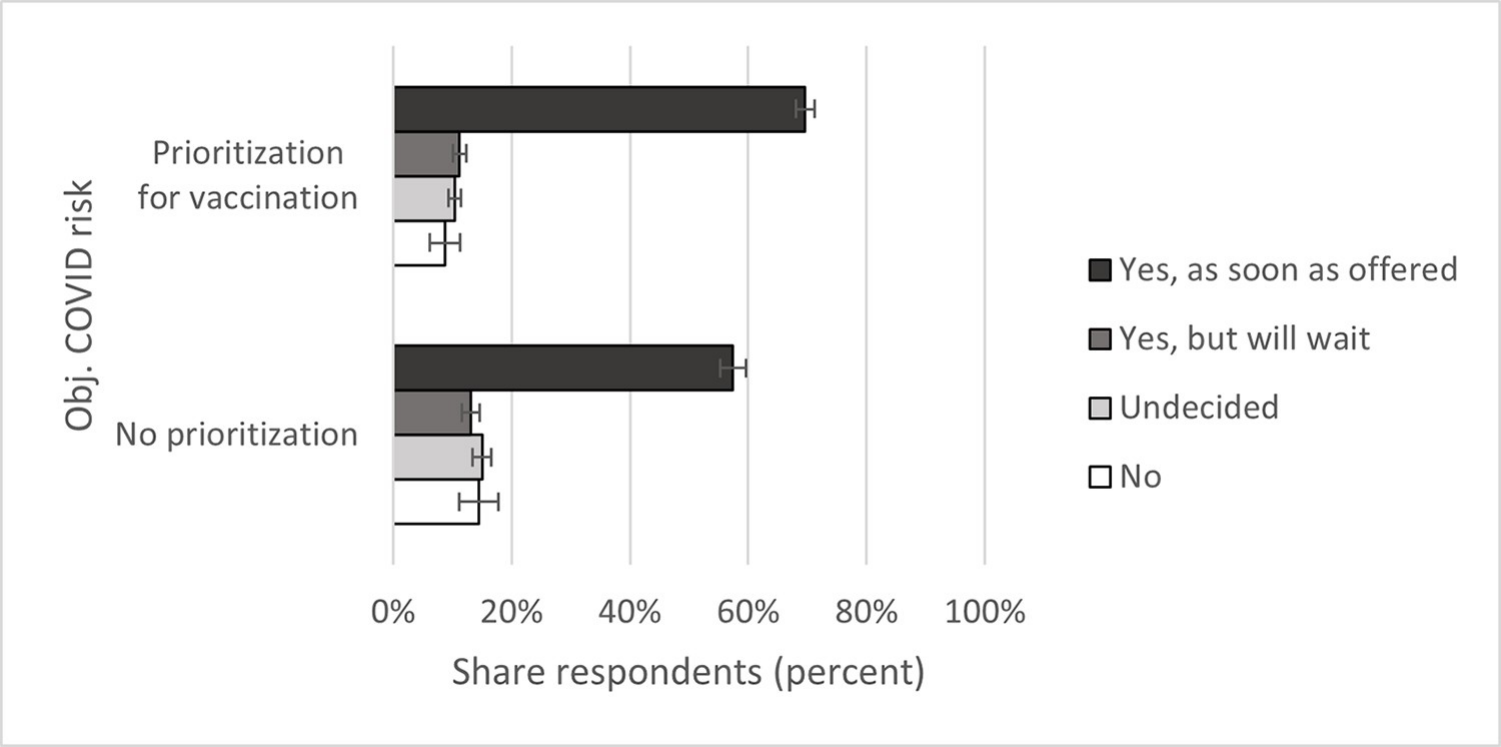
Official COVID risk and vaccine acceptance. Data: SOEP-CoV survey. Own calculations, weighted. 95% confidence intervals.

As expected, subjective and official COVID risk are generally positively connected to vaccine acceptance. Regarding subjective COVID risk (Fig 2), only respondents in the lowest risk quartile differed substantially in the “as soon as offered” category: 58 percent of those at the lowest risk of COVID chose this option, compared to 67 to 69 percent in the other quartiles. Concerning official COVID risk, respondents with heightened risk were with 70 percent more willing to get vaccinated as soon as offered compared to the 57 percent of respondents without heightened risk (Fig 3).

### Multiple regression analyses

To further gauge the relevance of generalized trust, we calculated Models 1–5 (Table 2) using multiple logistic regression with the dependent variable “vaccine acceptance,” for which 0 indicated vaccine hesitancy and 1 vaccine acceptance. We present the results as marginal effects, thus representing percentage point changes in the predicted probability to be vaccine-accepting.

**Table 2:** Multiple logistic regression—dependent variable: Vaccine acceptance.

	Model 1		Model 2		Model 3		Model 4		Model 5	
	dy/dx	SE	dy/dx	SE	dy/dx	SE	dy/dx	SE	dy/dx	SE
**Generalized trust**	0.052**	0.016	0.054**	0.015	0.077**	0.021	0.056**	0.016	0.090**	0.027
**Subjective COVID risk:** life-threatening infection (percent)			0.001**	0.000	0.004*	0.002				
Generalized trust * subjective COVID risk					-0.001	0.001				
**Official COVID risk:** prioritization for vaccination							0.087**	0.022	0.243*	0.096
Generalized trust * official COVID risk									-0.054+	0.032
**Female**	-0.097**	0.018	-0.100**	0.018	-0.101**	0.018	-0.100**	0.019	-0.099**	0.019
**Age**	0.005**	0.001	0.005**	0.001	0.006**	0.001				
**Migration history**	-0.109**	0.025	-0.109**	0.025	-0.109**	0.025	-0.132**	0.024	-0.133**	0.024
**Marital status**										
*Ref*: *Never Married*										
Married/Civil union	0.024	0.025	0.020	0.025	0.020	0.024	0.082**	0.025	0.083**	0.025
Divorced/Widowed	-0.026	0.030	-0.024	0.030	-0.024	0.030	0.053+	0.029	0.054+	0.029
**Education**										
*Ref*: *Max*. *lower secondary edu*.										
Intermediate secondary edu.	0.029	0.025	0.030	0.025	0.030	0.025	0.016	0.025	0.017	0.025
Upper secondary edu.	0.139**	0.034	0.146**	0.033	0.146**	0.033	0.115**	0.033	0.115**	0.033
Tertiary edu.	0.119**	0.029	0.127**	0.029	0.125**	0.029	0.111**	0.029	0.111**	0.029
**Household net equivalent income**	0.000*	0.000	0.000*	0.000	0.000*	0.000	0.000**	0.000	0.000**	0.000
**Occupational status**										
*Ref*: *Working*, *full-time*										
Working, not full-time	0.012	0.025	0.011	0.025	0.012	0.025	0.014	0.026	0.015	0.026
Not working	0.020	0.027	0.023	0.027	0.023	0.026	0.071**	0.026	0.072**	0.026
**Political leaning**										
*Ref*: *Left*										
Center	-0.034+	0.020	-0.033	0.020	-0.033	0.020	-0.038+	0.021	-0.037+	0.021
Right	-0.067*	0.027	-0.069*	0.027	-0.070**	0.027	-0.064*	0.028	-0.063*	0.028
**Satisfaction Fed. government**	0.050**	0.004	0.049**	0.004	0.049**	0.004	0.052**	0.004	0.052**	0.004
**Information source COVID**										
*Ref*: *Only traditional media*										
Mixed	0.004	0.020	0.003	0.020	0.003	0.020	-0.027	0.020	-0.027	0.020
Only Internet	-0.073*	0.036	-0.068+	0.037	-0.066+	0.037	-0.122**	0.036	-0.123**	0.036
N	5341		5341		5341		5341		5341	

Data: SOEP-CoV survey. Own calculations, weighted.

+ p < 0.10

* p < 0.05

** p < 0.01.

Model 1 contains the generalized trust index alongside the controls. As expected, generalized trust is positively significantly connected to vaccine acceptance: The more a respondent trusts others in general, the more likely are they to accept the vaccine. The marginal effect is moderately sized: Following the model and holding all controls constant, changing the trust index by one point on its 1 (lowest trust) to 4 (highest trust) scale is connected to a 5 percentage point change in the predicted acceptance probability.

In Models 2 and 3, the role of subjective COVID risk is studied. Model 2 adds the indicators for subjective COVID risk to the controls and the generalized trust index. In line with expectations, higher subjective risk is significantly positively connected to vaccine acceptance. However, the small effect size of subjective COVID risk is surprising: For example, respondents who estimate their risk of contracting a life-threatening COVID infection within the next 12 months at 50 percent are estimated to be only around 5 percentage points more likely to be vaccine-accepting than those who estimate their risk at 0 percent.

For Model 3, an interaction term was added to the subjective COVID risk indicator, the generalized trust index, and the controls. This term is an interaction variable between subjective COVID risk and the generalized trust index. However, the interaction term does not reach significance. This indicates that, contrary to expectations, the role of generalized trust in vaccination acceptance is comparable between individuals with different subjective COVID risk assessments.

In Models 4 and 5, the role of officially defined COVID risk is studied. In Model 4, the indicator for official COVID risk is added to the trust indicator and the controls. Following expectations, higher official COVID risk is connected to higher vaccine acceptance: holding other factors constant, respondents at heightened COVID risk are around 9 percentage points more likely to be vaccine accepting compared to those without heightened risk.

In the final Model 5, an interaction term was added to the generalized trust index, the official risk indicator, and controls. In line with our expectations, the coefficient for the main effect of generalized trust which refers in Model 5 to individuals in the lowest priority group, increases considerably in size compared to the previous model, which reports the mean trust-effect in the sample. The indicator for heightened COVID risk is significantly positive. As expected, respondents without heightened risk experience an especially strong link between generalized trust and vaccine acceptance: for this group, the predicted probability of vaccine acceptance increases by 9 percentage points with every point increase in the trust index value. The respective increase is around 5 percentage points smaller for respondents with heightened COVID risk. However, this interaction term is only marginally significantly negative.

## Summary and discussion

In this article, building on the idea that vaccine acceptance is embedded in the societal context, we studied the role of generalized trust in COVID-19 vaccine acceptance. Employing data from the German SOEP-CoV survey collected in early 2021, we found a significant positive link between respondents’ generalized trust and their willingness to be vaccinated as soon as the possibility of vaccination was offered to them. We theorize that generalized trust is one way to alleviate the collective action problem inherent in mass vaccination campaigns: Individuals who are generally trusting in others are more confident that collective efforts in times of crisis will succeed and are more willing to act to help strangers.

Furthermore, we theorized that the motivating force of generalized trust is most pronounced in respondents with a low personal COVID risk, as personal risk is lacking as a motivator for this group. By contrast, respondents with a high COVID risk may already be sufficiently motivated by self-interest. However, we find no clear support for this hypothesis.

We propose that the positive link between generalized trust and vaccine acceptance is rooted in the collective action problem. However, another theoretical explanation seems also plausible: as the general public is unable to judge the safety and efficacy of vaccines on its own, it has to trust the judgement of vaccine providers (the healthcare professionals administrating the vaccine) and policy makers (the health system, government, and researchers involved with the vaccine) [17]. Such forms of institutional trust and generalized trust may be connected on the individual level: it has been argued [54, 64] that experiences of (un)trustworthiness within institutions are generalized towards other citizens as well, thus spilling over into generalized trust. Likewise, certain individual dispositions towards (dis)trust may influence both institutional and generalized trust [64]. Although this view likewise proposes that persons high in generalized trust should be more vaccine accepting, it would also hypothesize that this link is at least partly mediated by certain forms of institutional trust.

Unfortunately, we were not able to control for additional forms of institutional trust, as no relevant items were available in the dataset. If generalized trust and one or more of the omitted forms of trust are indeed correlated, this may lead to biased estimations for the effect of generalized trust. However, we were able to control for “satisfaction with the federal government’s crisis management” as a proxy for government trust. [19] found that generalized trust has an independent effect on vaccine acceptance when controlling for institutional trust in health care. [25] could show that the positive effect of generalized trust is partly mediated by trust in the healthcare system, but has also an independent effect. Future research may be able to include both generalized trust and a wider set of institutional forms of trust in their analyses and thus be able to disentangle both theoretical mechanisms.

We do not find heterogeneous effects for generalized trust between different subjective COVID risk groups. Instead, the link is of similar strength when comparing respondents with different assessments of how likely a life-threatening COVID infection is for them personally. Regarding the official risk measure based on age and health conditions, risk groups only differ on a marginally significant level concerning the link between generalized trust and vaccine acceptance.

It may be argued that the missing interaction effect between subjective COVID risk and generalized trust indicates that there are actually no heterogeneous effects according to COVID risk. Instead, the interaction effect between official COVID risk and generalized trust may have another explanation: The second wave of the SOEP-CoV survey was conducted when the German vaccination campaign was just beginning. At this point in time, according to the published priority plan, only older people, individuals with preexisting health conditions, and workers in certain (frontline) occupations were offered vaccination immediately. Thus, only this group was confronted with a vaccination decision in the near future, while individuals with low priority did not have to think about the question yet. The differing answering patterns regarding the vaccination question in the survey could therefore have been due to this difference in the real-world decision process and not to lower or higher self-interest in vaccination. To test this idea, we estimated an additional multiple regression model similar to those presented in Table 2. In this model, we included an interaction term between working in a prioritized occupation (e.g., frontline medical worker) and generalized trust. We assumed that respondents in these prioritized occupations would have been confronted with a decision on vaccination in the near future but that they did not necessarily differ in self-interest in vaccination from other respondents. We did not find a significant interaction effect between being in a prioritized occupation and generalized trust. Therefore, we do not have empirical evidence that the timeframe of vaccination access was responsible for the reported results.

Another explanation for lacking differences according to subjective COVID risk may be low validity of the employed item. With an average value of 23 percent in the entire sample, respondents dramatically overestimated their personal risk of life-threatening COVID disease within the next 12 months. Members of the SOEP group point out that this may be due to the general psychological tendency to overestimate the probability of highly unlikely life-threatening events, and to the fact that many respondents picked “50 percent” to express their uncertainty [65]. The relatively weak link between subjective COVID risk and vaccine acceptance stands in stark contrast to the high probability of life-threatening disease indicated by some respondents: For example, respondents choosing a probability of 50 percent were only 5 percentage points more vaccine-accepting than those estimating a risk of 0 percent, holding other factors constant.

Official COVID risk groups differ on a marginally significant level regarding their link between generalized trust and vaccine acceptance. According to expectations, the link is stronger for the lower-risk group. However, although one of the most important risk factors for serious COVID consequences is advanced age, we cannot outrule that non-risk related factors linked to older age may be responsible for this result.

In conclusion, our analyses do not sufficiently support the idea that there are heterogeneous effects of generalized trust on vaccination acceptance according to COVID risk. However, additional research employing possibly more valid COVID risk indicators to reexamine this result would be very welcome.

Our findings indicate that generalized trust is an important resource on the individual level to enable action for the common good in times of crisis.

## Supporting information

S1 Table Detailed variable distribution: Components of official COVID risk. Data: SOEP-CoV survey. Own calculations, weighted.
(DOCX)
