# Supplementary material for: The role of generalized trust in COVID-19 vaccine acceptance
Source: PLoS One. 2022 Dec 22;17(12):e0278854. doi: 10.1371/journal.pone.0278854 (PMC9779038; doi:10.1371/journal.pone.0278854)
Supplement: S1 Table — Data: SOEP-CoV survey. Own calculations, weighted. (DOCX) [file pone.0278854.s001.docx]

|  | Mean |
| --- | --- |
| **Official COVID risk:** governmental prioritization for vaccination | 0.65 |
| **Age 60 and older** | 0.67 |
| **Obese** (BMI >=30) | 0.30 |
| **Relevant health condition** (present or past) | 0.70 |
| **Dementia** | 0.00 |
| **Heart disease** | 0.26 |
| **Diabetes** | 0.21 |
| **Cancer** | 0.14 |
| **Hypertension** | 0.69 |
| **Asthma** | 0.18 |
| N | 5341 |
